# Performance of Pheromone-Baited Traps to Monitor the Seasonal Abundance of Tortrix Moths in Chestnut Groves

**DOI:** 10.3390/insects11110807

**Published:** 2020-11-17

**Authors:** Chiara Ferracini, Cristina Pogolotti, Giada Lentini, Valerio Saitta, Enrico Busato, Franco Rama, Alberto Alma

**Affiliations:** 1 Department of Agricultural, Forest and Food Sciences (DISAFA), University of Torino, Largo Paolo Braccini 2, 10095 Grugliasco, Italy; cristina.pogolotti@unito.it (C.P.); giada.lentini@unito.it (G.L.); valerio.saitta@unito.it (V.S.); enrico.busato@unito.it (E.B.); alberto.alma@unito.it (A.A.); 2 Biological Products Unit, Isagro S.p.A., Via Fauser, 28, 28100 Novara, Italy; franco.rama24@gmail.com

**Keywords:** pheromone-baited trap, chestnut tortrix moth, *Castanea sativa*, male genitalia, carpophagous insect pests

## Abstract

**Simple Summary:**

Investigations were performed in 2018–2019 in chestnut groves in northern Italy to monitor the seasonal flight activity of *Pammene fasciana* (L.), *Cydia fagiglandana* (Zeller), and *C.* *splendana* (Hübner) with pheromone-baited traps. Commercially available and experimental pheromone blends were tested. Newly formed chestnut husks and fruits were randomly collected to evaluate damage. Damage was correlated with trap catches. *P. fasciana* was present in all the sites, while *Cydia* species were recorded in three of six sites, with differences in abundance related to pheromone blends studied. Several morphologically similar non-target species occurred, highlighting the risk of overestimating catches. Fruit damage did not correlate with trap captures, suggesting that monitoring probably underestimates the true size of the moths’ populations. These data contribute to ascertaining the presence of tortrix moths in northern Italian chestnut groves, and are important for planning specific control measures.

**Abstract:**

(1) *Background*: *Pammene fasciana* (L.), *Cydia fagiglandana* (Zeller), and *C. splendana* (Hübner) (Lepidoptera: Tortricidae) are considered key moth pests of chestnut in Europe. (2) *Methods*: Investigations were performed in 2018–2019 in northern Italy. Sticky traps and commercially available pheromones were used for monitoring; moreover, two experimental pheromone blends were tested. All specimens were identified according to male genitalia and molecular analyses. Newly formed chestnut husks and fruits were randomly collected to evaluate the presence of larvae and/or feeding damage, by comparing it to trap catches. (3) *Results*: *P. fasciana* was present in all the sites, whereas *Cydia* species were recorded in three sites of six, with differences in abundance related to pheromone blends studied. Several non-target species, such as *Oegoconia novimundi* (Busck) and *Cydia ilipulana* (Walsingham), were present. Data about the seasonal flight activity are provided. (4) *Conclusions*: This research contributes to ascertaining the presence and abundance of tortrix moths in Italian chestnut groves, and the presence of non-target species highlights the risk of overestimating catches. Fruit damage recorded did not always reflect catches made by pheromone traps, suggesting that monitoring may underestimate the real size of moths’ populations. All of the data acquired are important for planning specific control measures.

## 1. Introduction

Three species of carpophagous tortricids are responsible for significative yield losses in chestnut production, namely *Pammene fasciana* (L.), *Cydia fagiglandana* (Zeller), and *C. splendana* (Hübner) (Lepidoptera: Tortricidae) [1,2,3,4]. These are considered the key moth pests of chestnut in Europe, but they also share other common host plants. Indeed, north of the geographical distribution of chestnut, the larvae of *C. fagiglandana* feed on beech nuts (*Fagus sylvatica* L.) and the larvae of *C. splendana* on acorns (*Quercus* spp.). In southern Europe, both species are also found on sweet chestnut (*Castanea sativa* Miller). All of these moths are oligophagous and monovoltine, with fruit feeding (carpophagous) larvae developing in the fruit. The larvae, with their trophic activity, cause premature drops of fruits, destruction of the cotyledons, and reduction in weight and size. They are responsible for extensive economic losses annually, with fruit losses up to 70% of harvested fruits depending upon the year, plantation, and geographical region [5]. In many European countries and sites, *P. fasciana* is considered to be a minor chestnut feeding pest; conversely, the congeneric species *C. fagiglandana* and *C. splendana* have received the most attention.

Controlling the moths with pesticides is difficult due to the endophytic development of the larvae. Moreover, because these species commonly co-occur and co-infest chestnut orchards, a simultaneous monitoring plan is highly desirable. In this context, many insecticidal applications may fail, because one species can have a population outburst when the orchard is not covered by insecticide in terms of timing. The potential of sex attractants and plant volatiles for practical application in chestnut moth control has been investigated [6,7,8]. The most important application of pheromones is for monitoring purposes, to evaluate if the target species is present or absent in an area or to determine if enough insects are present to warrant a costly treatment. Population monitoring is an excellent tool for monitoring pest populations in surveys, and is considered the keystone of integrated pest management [9]. Sticky traps baited with sex attractants are commonly used to investigate flight activity and monitor seasonal flight population dynamics. Furthermore, the use of sex pheromones in specific control programs, such as mating disruption, are the most effective measures to control these carpophagous insect pests of chestnut in Europe. Recently, a novel approach using an innovative pheromone-dispensing device in the form of “puffers” has also been tested [1,3,10], although further investigations are still needed. However, before implementing any control program, target species must be identified and their population dynamics investigated. With regard to the chestnut tortrix moths, damage is reported in the literature as variable in different years and sites. Chestnuts may be heavily attacked in some years and localities, whereas at other times infestation is reported to be scattered. In particular, due to the similarity in morphological traits and behavior, these moths often cannot be reliably separated to species; hence, an accurate identification is essential before applying any control strategy. Most procedures rely on morphological characterization of male genitalia and on molecular-based approaches. Nuclear ribosomal DNA (rDNA) and mitochondrial DNA (mtDNA) have been widely used for taxonomic and phylogenetic studies in insects, including tortricids, and thus may represent a rapid and reliable method to discriminate among species [11,12,13].

The distribution and prevalence of these tortrix moths in chestnut orchards has been investigated in France and Switzerland [14], Greece [11], Hungary [15], and Portugal [16,17]. With regard to Italy, investigations have been performed in northern Italy [1,18], central Italy [4], and southern Italy [19]. The actual distribution of these species has not been investigated in detail in other northern regions, specifically north-western Italy. Our research objectives were to assess the species richness and phenology in chestnut groves, using pheromone-baited traps, with the aim of acquiring new data essential for improving environmentally friendly control strategies. In particular, our aim was to (a) identify the species responsible for infestations in different Italian regions; (b) investigate their populations’ dynamics; (c) evaluate the effectiveness of different pheromone lures (commercial and experimental) for monitoring *Cydia* spp. moths; and (d) compare trap catches to damage recorded in chestnut husks and fruits.

## 2. Materials and Methods

Investigations were performed over a two-year period (2018–2019) in sweet chestnut groves located in six sites in four Italian regions, namely Emilia-Romagna, Liguria, Piedmont, and Tuscany (Figure S1; Table S1). The investigated chestnut plants were unmanaged, with productive *C. sativa* trees generally 80–120 years old. The chestnut trees in all sites were grassed with herbaceous monicotyledons and dicotyledons, and surrounded by various woody broadleaf species (oaks, hophornbeam, wild cherries, maples, and ashes).

### 2.1. Monitoring Traps

The population dynamics of *P. fasciana, C. fagiglandana*, and *C. splendana* were investigated using sex pheromone lures produced by Isagro S.p.A. Monitoring was carried out in both years from May to October. Commercially available pheromones were used for monitoring *P. fasciana* (Z8-12:Ac + Z8-12OH), *C. fagiglandana* (E8,E10-12Ac), and *C. splendana* (E8,Z10-12:Ac). Moreover, two additional synthetic experimental pheromone blends were tested, for *C. fagiglandana* (E8,E10-12Ac), and *C. splendana* (Tricarbonyl-[(8,9,10,11-η)-8,10-dodecadien-1-yl acetate]-iron) (Table 1). Pheromone dispenser capsules were placed in the center of the sticky surface of delta traps (Traptest Isagro^®^, Novara, Italy), and in each site three replicates of each lure were used. Traps were attached to chestnut branches at heights of 5–6 m, in the outer surface of the tree canopy. Traps were spaced 50 m apart and at least 20 m from the border of the plot. Pheromone lures were replaced every four weeks, whereas traps were inspected weekly. At each inspection the sticky liner of the trap was removed and replaced with a new one. The position of the traps was interchanged regularly to avoid the position effect. All of the sticky surfaces removed were taken to the laboratory in order to count and identify adult moths. Any non-lepidopteran insects were discarded.

For convenience, commercial and experimental pheromones lures will be hereafter referred to as CP and EP, respectively. Therefore, all over the manuscript traps are shown as PF traps (CP), CF traps (CP), or CS traps (CP) with regard to the commercially available pheromones traps for *P. fasciana, C. fagiglandana*, and *C. splendana*, respectively. Moreover, CF traps (EP) and CS traps (EP) are used in the case of experimental pheromone-baited traps for *C. fagiglandana* and *C. splendana,* respectively (Table 1).

### 2.2. Fruit Collection

In 3 sites out of 6 (Villar Focchiardo, Montese, and Badia del Borgo), a sample of newly formed chestnut husks was picked up after the physiological drop. In each site a minimum of 25 husks was randomly collected in 4 different plots. All of the fruits were taken to the laboratory and dissected with a scalpel for checking the presence of damage by *P. fasciana* (larvae, feeding damage, frass).

Moreover, a sample of at least 800 chestnut fruits per site was randomly hand-picked from the ground three times during the fruit fall (late September, early October, and mid-October). In each site and for each collection period, a minimum of 60 fruits was randomly collected in 4 different plots. Nuts were visually inspected after collection for the presence of exit holes, then stored in cardboard boxes in outdoor conditions, and checked daily to record the number of newly emerged larvae. Only tortricid larvae were considered, and any larva belonging to the chestnut weevil *Curculio* spp. (Coleoptera: Curculionidae) and/or any emergence hole with a typical diameter attributable to *Curculio* spp. (>2.5 mm) was discarded. Stored fruits were checked daily until no emergence was recorded for three consecutive weeks.

### 2.3. Identification of Specimens

A representative sample corresponding to 70% of the total number of males caught in pheromone traps was morphologically identified according to the male genital structures. The sample was chosen on the basis of year, site, target species, pheromone, trap replicate, and survey week. The abdomens of specimens were cut off and boiled in 10% KOH. The rigid genitalia were then rinsed with distilled water and mounted on microscope slides. The scales adhering to the abdomen pelt were cleaned using a very thin brush. The male clasping genital structures, valvae, were spread in a standard position across all specimens. The male genitalia were photographed using a Leica DFC290 digital camera mounted on a Leica MZ16A stereomicroscope (40× magnification) and processed with the Leica application suite V3.7 software (Leica Microsystems, Wetzlar, Germany). Species were identified according to male genitalia following the keys by Razowski [21], and/or compared with voucher specimens deposited at the DISAFA-Entomology laboratory, Italy.

The larvae obtained from chestnut fruits were identified based on morphological traits and type of damage [22], and/or compared with voucher specimens deposited at the DISAFA-Entomology laboratory. The remaining males caught in pheromone traps, doubtful adult specimens (characterized by different size, forewing color, and/or forewing pattern), and a sample corresponding to 20% of the total number of larvae recorded (chosen on the basis of year, site, fruit fall period) were submitted for DNA extraction according to the method described by Asghar et al. [23], and then sequenced for the cytochrome oxidase I (COI) gene following Hajibabaei et al. [24]. The sequences were compared with those in the National Centre for Biotechnology Information sequence database.

### 2.4. Statistical Analysis

After testing for homogeneity of variance (Levene’s test), data were analyzed using the Student’s *t* tests (*p* < 0.05) to compare the different pheromone blends (commercial and experimental) for *C. fagiglandana* and *C. splendana*. Weekly trap counts were pooled for each site and year. Data that did not exhibit a normal distribution or homogeneity of variance were log (*x* + 1) transformed.

To determine the relationship between the number of male moths caught on the traps and the number of larvae recorded in damaged husks and chestnut fruits, Pearson’s linear correlation coefficient (r) was calculated, and its significance was estimated at *p* < 0.05.

A two-way ANOVA was performed to analyze the damage, with the number of larvae recorded as the response variable and species and sites as main effects. A post hoc analysis using Tukey’s all-pair comparisons was performed, with a significance level set at 0.05.

All analyses were performed using the software SPSS version 20.0 (SPSS, Chicago, IL, USA).

## 3. Results

### 3.1. Identification of Specimens

**Figure 1:**
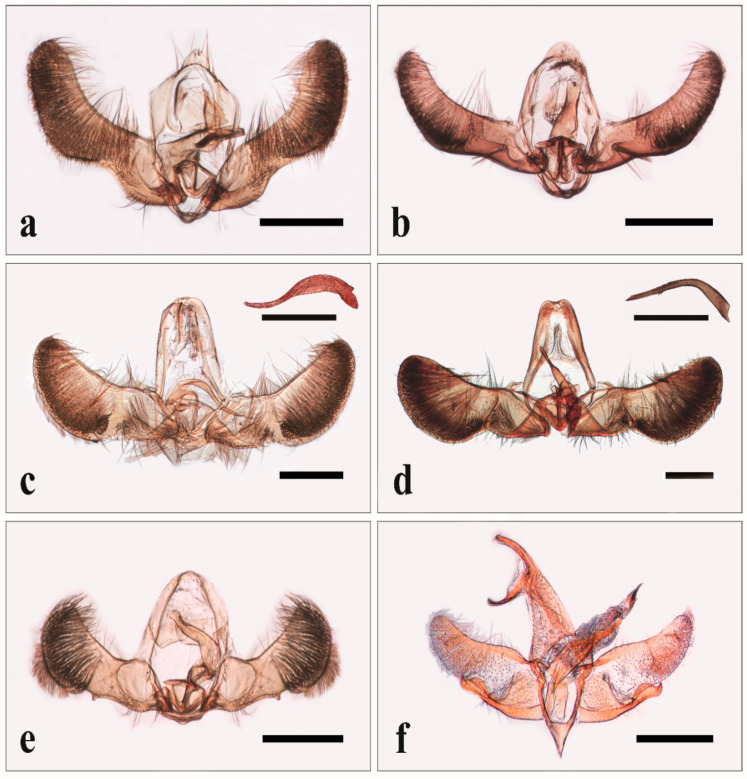
Male genitalia of: *Pammene fasciana* (L.) (**a**); *P*. *suspectana* (Lienig & Zeller) (**b**); *Cydia fagiglandana* (Zeller) with *aedeagus* in lateral view (**c**); *C*. *splendana* (Hübner) with *aedeagus* in lateral view (**d**); *Grapholita* (*Aspila*) *funebrana* Treitschke (**e**); *Oegoconia novimundi* (Busck) (**f**). Bar: 500 μm.

**Figure 2:**
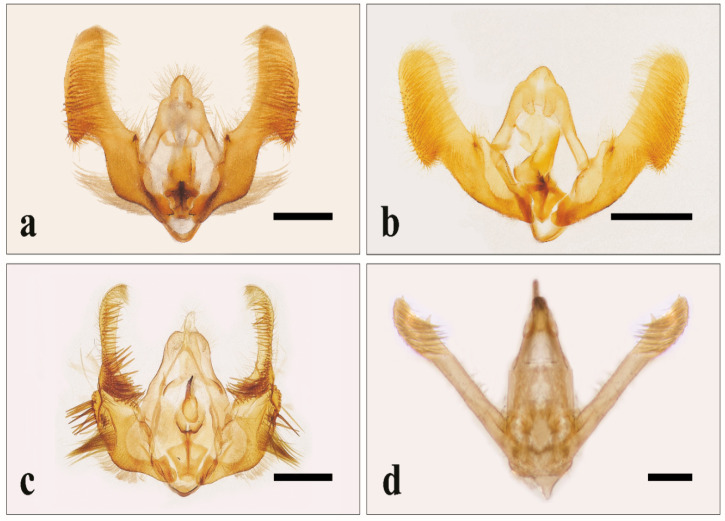
Male genitalia of: *Epiblema foenella* (L.) (**a**); *E*. *scutulana* (Denis & Schiffermüller) (**b**); *Celypha striana* (Denis & Schiffermüller) (**c**); *Idaea rusticata* (Denis & Schiffermüller) (**d**). Bar: 500 μm.

A total of 3588 and 6884 individuals were passively caught in traps over the sampling period between May and October in 2018 and 2019, respectively. The pictures of the male genitalia of the species most frequently found are reported in Figure 1 and Figure 2.

**Figure 3:**
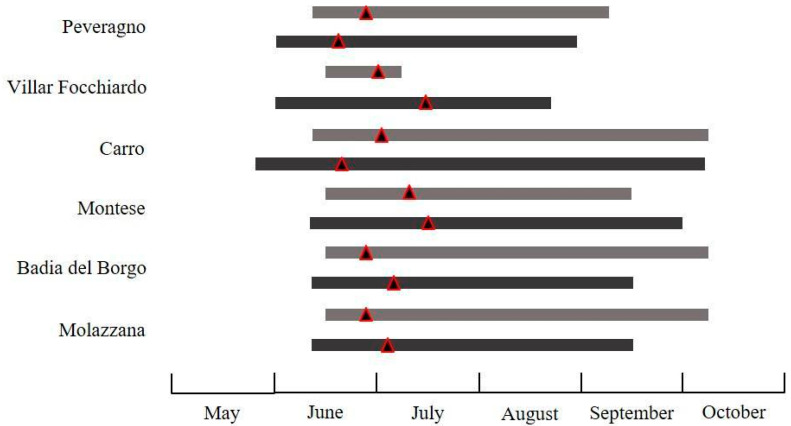
Seasonal flight activity of *Pammene fasciana* (L.) recorded in the surveyed sites using the commercially available pheromone (grey bars refer to 2018, black bars to 2019. For each bar, the triangle corresponds to the population peak).

**Figure 4:**
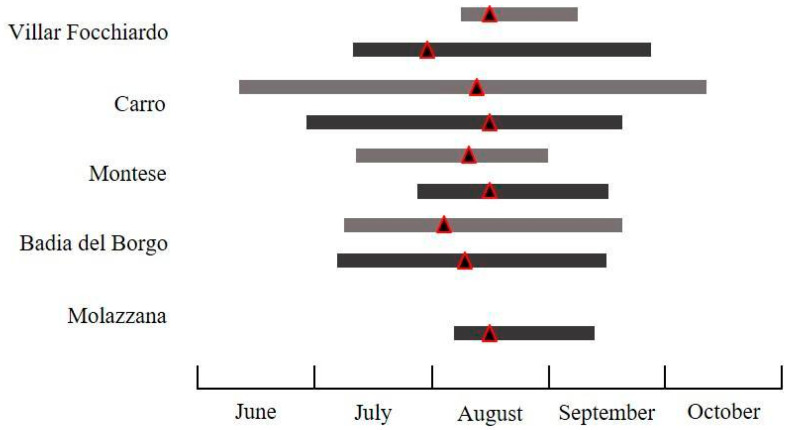
Seasonal flight activity of *Cydia fagiglandana* (Zeller) recorded in the surveyed sites using the commercially available pheromone (grey bars refer to 2018, black bars to 2019. For each bar, the triangle corresponds to the population peak).

**Figure 5:**
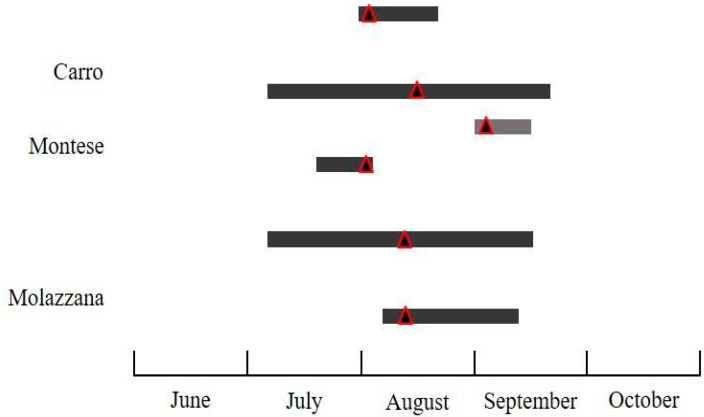
Seasonal flight activity of *Cydia fagiglandana* (Zeller) recorded in the surveyed sites using the experimental pheromone (grey bars refer to 2018, black bars to 2019. For each bar, the triangle corresponds to the population peak).

**Figure 6:**
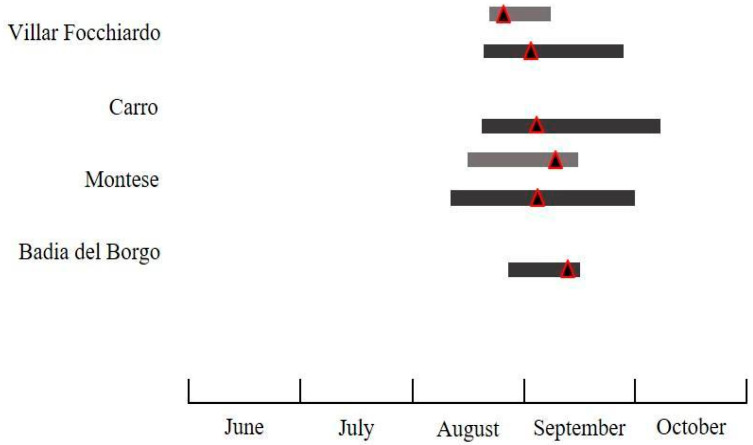
Seasonal flight activity of *Cydia splendana* (Hübner) recorded in the surveyed sites using the experimental available pheromone (grey bars refer to 2018, black bars to 2019. For each bar, the triangle corresponds to the population peak).

**Figure 7:**
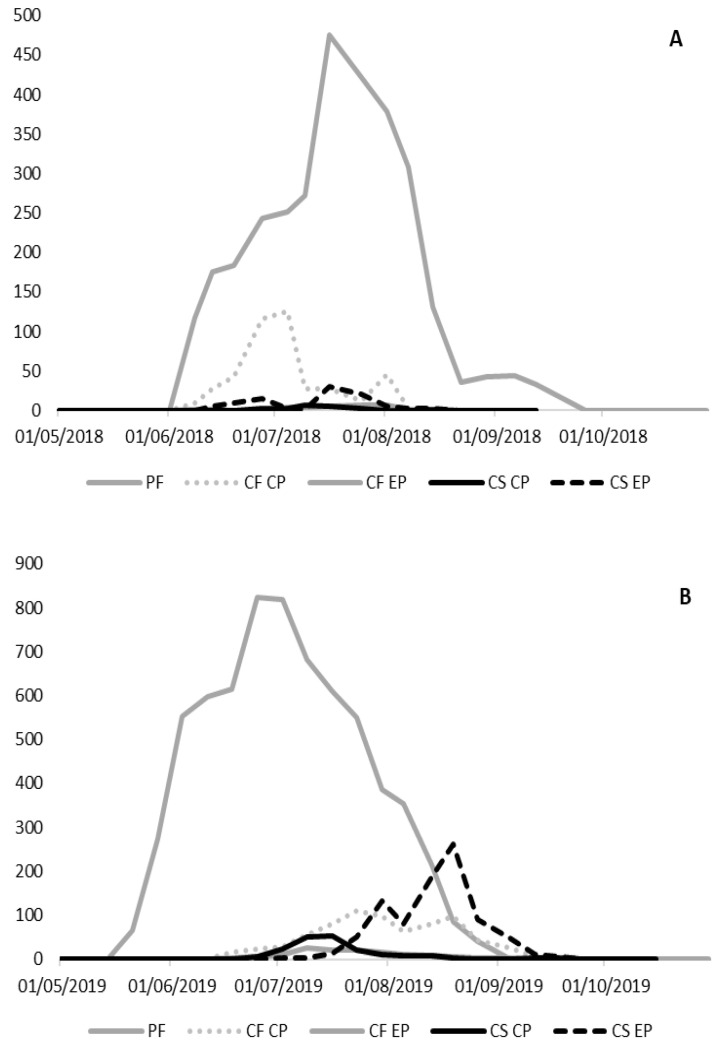
Total number of male moths recorded in all the surveyed sites in 2018 (**A**) and in 2019 (**B**) (PF, *Pammene fasciana* (L.); CF, *Cydia fagiglandana* (Zeller); CS, *Cydia splendana* (Hübner); CP, commercial pheromone; EP, experimental pheromone).

The flight activity of the investigated species displayed differences in abundance (Table 2) and temporal patterns of flight activity in the different surveyed regions and years. The seasonal flight activity and the total number of male moths captured by traps for all species, pheromone blends, sites, and years is reported in Figure 3, Figure 4, Figure 5, Figure 6 and Figure 7.

### 3.2. Pheromone Experiment

#### 3.2.1. *Pammene fasciana*

The results of the trials showed that *P. fasciana* is a common species, present in all the surveyed sites, whereas *C. fagiglandana* and *C. splendana* were less abundant, being recorded especially in three sites of six (Villar Focchiardo, Carro, Montese, Italy). Most of the adults (3019 and 5298), representing 84.14% and 76.96% of the total specimens, respectively, were collected on PF traps in 2018–2019 (Table 2). According to the observation of the male genital structures and the molecular analyses, the individuals captured corresponded to several species, and *P. fasciana* accounted for about 62% of these. Several non-target species were recorded, namely *Celypha striana* (Denis & Schiffermüller), *Epiblema scutulana* (Denis & Schiffermüller), *Grapholita* (*Aspila*) *funebrana* Treitschke, *P. suspectana* (Lienig & Zeller) (Lepidoptera: Tortricidae), and *Oegoconia novimundi* (Busck) (Lepidoptera: Autostichidae).

The activity of *P. fasciana* started in late May-mid June, then the rate of captures in pheromone traps increased and reached a peak in late June-mid July, depending on the sites. This species was recorded in a limited time space, from mid-June to mid-July only in Villar Focchiardo in 2018. In all of the other sites and years, males exhibited a longer temporal pattern until September, and early October in four sites of six (Carro, Montese, Badia del Borgo, Molazzana) (Figure 3). The average number of individuals per trap ranged from 6.33 ± 0.33 to 163.33 ± 12.14.

*P. fasciana* represented the main species recorded in three sites (Carro, Molazzana, and Montese) of six in both years, whereas in Villar Focchiardo it accounted for 2% and 10%, in 2018 and 2019 respectively, with *O. novimundi* being the most abundant. The other non-target species, namely *C. striana*, *E. scutulana G. funebrana*, and *P. suspectana* were generally less abundant, and represented on average 2%, 7%, 9%, and 4% for all sites and years. Compared to PF traps, males trapped in CF and CS traps were less abundant. Higher catches were recorded with commercial pheromone for *C. fagiglandana* (12.12% of the total specimens collected, in 2018), and with the experimental pheromone for *C. splendana* (9.78% of the total specimens collected, in 2019) (Table 2). On the CF traps some *C. splendana* individuals were recorded and, vice versa, on CS traps some *C. fagiglandana* specimens were also recorded.

#### 3.2.2. *Cydia fagiglandana*

For *C. fagiglandana*, the number of males recorded comparing the commercial pheromone versus the experimental one differed significantly only in 2018 (*t* = 47.45, *df* = 1, 5, *p* = 0.031 for 2018; *t* = 28.35, *df* = 1, 5, *p* = 0.951). In total, CF traps (CP) recorded 824 individuals versus 402 individuals captured in CF traps (EC) in 2018–2019 (Table 2). In 2018 the species occurred in four out of six sites, with a higher presence in Badia del Borgo and Montese. In these sites, males were found from mid-June to mid-October, and registered the maximum flight activity in early-mid August (Figure 4 and Figure 5). The beginning and the end of the flight period displayed differences, and in several localities the first males were recorded about one month later. No *C. fagiglandana* adults were ever recorded in Peveragno, and in Molazzana no males were ever collected in 2018. The average number of individuals per trap ranged from 1.70 ± 1.67 to 17.0 ± 7.64. In CF traps (CP), *C. fagiglandana* represented the main species recorded in three sites of six in both years (Carro, Montese, Badia del Borgo), whereas in Villar Focchiardo it accounted for 32% and 38%, in 2018 and 2019 respectively, with *C. splendana* being the most abundant. In the same site, other non-target species, namely *Cydia duplicana* (Zetterstedt) (Lepidoptera: Tortricidae), and *Idaea rusticata* (Denis & Schiffermüller) (Lepidoptera: Geometridae), were recorded, representing 7% and 8%, respectively, in CF traps (CP). In CF traps (EP), only five individuals were recorded in Montese in 2018, whereas in 2019 *C. fagiglandana* accounted for more than 70% in four sites. The only non-target species recorded was *Cydia ilipulana* (Walsingham) (Lepidoptera: Tortricidae) accounting for 9%, 18%, and 25% in 2019 in Villar Focchiardo, Carro and Badia del Borgo, respectively.

#### 3.2.3. *Cydia splendana*

With regard to *C. splendana*, the trap catch data revealed that a significantly highest number of moths was trapped using the experimental pheromone instead of the commercial one, with CS traps (EP) recording 749 individuals versus 180 individuals captured in CS traps (CP) in 2018–2019 (*t* =21.77, *df* = 1, 5, *p* = 0.037 for 2018; *t* = 13.75, *df* = 1, 5, *p* = 0.019 for 2019) (Table 2). The species accounted on average for 62%, and was found in four sites of six, whereas in Peveragno and Molazzana, no *C. splendana* adults were ever recorded with both pheromones and in both years. The highest total number of male adults recorded in CS traps (EP) was 49 and 77 males, in 2018 and 2019, respectively. On average, adults were found from late August to mid-September, registering the maximum flight activity in late August. The average number of individuals per trap ranged from 0.67 ± 0.33 to 75.0 ± 3.21. Given the small number of individuals found in CS traps (CP), only the seasonal flight activity recorded in CS traps (EP) is reported (Figure 6).

In general terms, in CS traps (CP) very few *C. splendana* adults were recorded (on average less than 2 in all the survey period), and in some traps, *C. fagiglandana* individuals were higher in number (e.g., 49 males recorded in Carro in 2019). In some traps few individuals of the non-target species *Epiblema foenella* (L.) (Lepidoptera: Tortricidae), and *I. rusticata* were also recorded, but these all accounted for less than 1%.

#### 3.2.4. Damage to Chestnut Husks and Fruits

With regard to chestnut husks and fruits, the total number of healthy and damaged husks, and the total number of larvae emerging from chestnut fruits and emergence holes, were recorded. The percentage of damage is reported in Table 3 and Table 4. A high percentage of damaged husks was recorded, particularly in 2019, when more than half of the dissected newly formed husks showed signs of larvae, feeding damage, and/or frass in two sites of three (Table 3). In all sites, *P. fasciana* accounted on average for 91% of the larvae recorded, whereas *C. fagiglandana* and *C. splendana* accounted for 2% and 7%, respectively. Infestation of chestnut husks differed statistically when compared to species and sites in 2018 (among sites F = 3.30, *df* = 4, 67, *p* = 0.022; among species F = 9.33, *df* = 2, 67, *p* < 0.001) and in 2019 (among sites F = 5.59, *df* = 4, 67, *p* < 0.001; among species F = 17.66, *df* = 2, 67, *p* < 0.001). A significant positive correlation between males catches and damaged husks was found only in Liguria region (Pearson’s linear correlation, Carro: r = 0.341, *p* = 0.023), whereas there were no significant correlations for the other sites (Pearson’s linear correlation, Peveragno: r = 0.161, *p* = 0.390; Villar Focchiardo: r = −0.131, *p* = 0.489; Montese: r = −0.037, *p* = 0.876; Badia del Borgo: r = −0.240, *p* = 0.27; Molazzana: r = 0.0247, *p* = 0.429).

With regard to infested chestnut fruits, 96% of the larvae recorded were identified as *C. fagiglandana*, and the remaining 4% was identified as *C. splendana.* Infestations differed statistically when compared to sites and species in 2018 (among sites F = 2.99, *df* = 4, 25, *p* = 0.044; between species F = 2.29, *df* = 1, 25, *p* = 0.026) and 2019 (among sites F = 5.80, *df* = 4, 61, *p* < 0.001; among species F = 4.196, *df* = 1, 61, *p* = 0.045). In particular, the average number of larvae recorded was significantly higher in Carro and Villar Focchiardo in 2018–2019 (Tukey’s test, *p* < 0.05 in both cases). In the two-year period, in some sites there was a decline in damage, similar to the case for Villar Focchiardo and Carro; conversely, for the other sites the damage recorded increased in 2019 (Table 4). No significant correlation was ever found between male catches and infested chestnut fruits (Pearson’s linear correlation, Peveragno: r = 0.015, *p* = 0.295; Villar Focchiardo: r = −0.060, *p* = 0.533; Carro: r = 0.081, *p* = 0.815; Montese: r = −0.097, *p* = 0.647; Badia del Borgo: r = −0.203, *p* = 0.977; Molazzana: r = 0.0135, *p* = 0.655).

## 4. Discussion

In recent years, *P. fasciana* and *Cydia* spp. tortrix moths have become an increasing problem for chestnut growers, and the demand for meaningful risk assessment and useful methods to restrict damage is increasing. In this paper, the presence of chestnut tortrix moths was investigated in a two-year period to deepen knowledge about their population dynamics and phenology in northern Italy and, in particular, in Piedmont and Liguria regions, where no specific investigations with regard to monitoring have been performed to date.

*P. fasciana* was recorded in all of the surveyed sites, with higher population density occurring in Tuscany and Liguria regions (Badia del Borgo, and Carro); vice versa, very few individuals were recorded in Piedmont region (Villar Focchiardo). Actually, in several sites the number of males caught in PF traps was very high, but *P. fasciana* represented on average 62% of all of the individuals found (ranging from 6% in Villar Focchiardo to 99% in Carro). In fact, although pheromones are species specific, it has often been observed that several non-target insects are trapped, owing to faulty trap designs or attractive pheromone blends. In the literature, other moths belonging to different genera were caught in the trap [1,3]. In our investigation, non-target species were recorded with high frequency, with most species (*O. novimundi*, *G. funebrana*, and *P. suspectana*) accounting for up to 76% in the Piedmont region. *O. novimundi* accounted for 20% to 40% of the total of species recorded in Emilia-Romagna and Tuscany regions (Montese and Badia del Borgo), whereas other non-target species were minor. Most of the non-target species were collected on PF traps, conversely to Angeli et al. [1], who pointed out that the broader spectrum of catches concerned CF and CS traps.

*C. fagiglandana* was recorded in all of the surveyed sites, with the exception of Peveragno, in both years and with both pheromones, even if in less quantity than *P. fasciana*. The number of specimens per trap differed greatly between the commercial and experimental lures. In particular, most *C. fagiglandana* male adults were caught using the commercial pheromone (E8,E10-12Ac). Nevertheless, the same pheromone did not perform in the same way for all localities, suggesting that other factors in addition to pheromone composition may also be responsible. For instance, in Tuscany and Piedmont regions (Badia del Borgo, and Villar Focchiardo), no males were trapped with the experimental pheromone in 2018, whereas in 2019 the target species represented about 70% of the total males trapped.

On average, *C. fagiglandana* represented 69% of all of the individuals found (ranging from 32% in Villar Focchiardo in 2018 to 100% in Montese in 2018), and among non-target species *C. ilipulana* was the most abundant. Although *P. fasciana* exhibited similar temporal patterns in different sites and years, the seasonal flight activity for *C. fagiglandana* differed more markedly. In particular, in Carro in 2018 males started being caught almost a month earlier than in other sites. These differences are likely related to abiotic conditions, considering that Carro is the surveyed site located at the lowest altitudes, with the milder weather conditions here possibly favoring an earlier emergence of adults.

*C. splendana* was the least abundant of the three target species. Although in some localities it was a minor species, with extremely low captures (fewer than 10 individuals throughout the season), the highest male adult catches were with the experimental pheromone (Tricarbonyl-[(8,9,10,11-η)-8,10-dodecadien-1-yl acetate]-iron (1)).

In general terms, the three target species populations overlapped during the season, demonstrating a similar temporal pattern to previous investigations already carried out in NE Italy [1,25,26], even if in our investigations all three species exhibited a longer flight activity until early-mid October in some sites.

The knowledge of the seasonal flight patterns and spatial distributions contributes to ascertaining the presence of tortrix moths in northern Italian chestnut groves and better understanding of their population dynamics. Nonetheless, reliably linking catch number with absolute pest density across a growing season may be difficult, in particular if the pest distribution considerably varies through time and with particular geography [27]. Blomefield and Knight et al. [28] pointed out how the information provided by pheromone traps is not always easy to interpret. Factors such as moth density, immigration, temperature, moonlight, wind speed, trap and lure placement and maintenance, and competition between traps and calling females, can deeply affect the number of male moths caught in traps. The location of the trap within the canopy tree may represent a critical factor, as already demonstrated for codling moth [29], deeply influencing the likelihood of capturing moths in an environment in which male orientation to pheromone sources is severely impeded. In particular, in chestnut orchards higher catches were also recorded in the upper (4–8 m) compared to lower canopy positions for *C. fagiglandana* and *C. splendana* [1,25]. Local environmental conditions, in concert with interspecific interactions, could significantly alter the relative abundance of the target species. In particular, the differences in abundance recorded in the two-year period suggest the need to constantly monitor the occurrence and distribution of these pests, even of those described in the literature as minor (e.g., *P. fasciana*). The results of this study corroborate the findings previously reported by Avtzis et al. [11], who highlighted that it would be imprudent for any integrated pest management strategy to focus solely on one tortrix while ignoring others.

Moreover, being aware of the presence of non-target species in the traps highlights the magnitude of the risk of overestimating catches. All of the different species trapped, most of which belong to the Tortricidae family, can be easily confused with each other, leading to a possible misinterpretation of the presence of tortrix moths in chestnut orchards, thus showing the importance of the male genitalia observation and molecular analyses as a diagnostic character to make accurate identifications and appropriate control decisions. It is not surprising to find *C. fagiglandana* specimens in CS traps and vice versa, given the similarity of the pheromone blends, as previously noted by Pedrazzoli et al. [26].

With regard to chestnut newly formed husks, a positive correlation between males catches and damaged husks was found only in Liguria region, where the infestation recorded by *P. fasciana* and the percentage of damaged husks reached high levels. Conversely, in the Piedmont region, although a low infestation was recorded, 48.5% and 73.8% of husks were damaged in Peveragno and Villar Focchiardo in 2019, respectively. Concerning chestnut fruits, most *Cydia* larvae emerged in the two months following the harvest. Previous investigations of the chestnut weevil *C. elephas* (Gyllenhal) had, in fact, highlighted how the estimation of the infestations by visual assessment of the nuts immediately following collection is not adequate to estimate the true extent of infestation by the pests [30]. Our data showed that the infestation rate recorded at the end of the storage period (late December) increased about 2.3 times from that observed immediately following collection, with a maximum increase of 8 times recorded in Peveragno in 2019.

In addition, attempts to positively correlate trap catches with insect damage were not successful. Most *Cydia* spp. larvae emerged from chestnut fruits collected in Liguria region (Carro). In this site the abundances of *C. fagiglandana* and *C. splendana* were similar to those recorded in Tuscany and Emilia-Romagna regions (Badia del Borgo and Montese), where low infestation rates were found instead. In particular, in 2019 in Montese the infestation level of 12.14% was very high compared to the total number of adult males collected throughout the season (5 *C. fagiglandana* and 24 *C. splendana*). Thus, the damage recorded does not always reflect catches made by pheromone traps, suggesting that monitoring may underestimate the real size of the moths’ populations. Sieber et al. [30] reported that the size of the fruits had a statistically significant effect on colonization by *C. splendana*, unlike chestnut variety and harvest (net versus ground). Conversely, harvest and size clearly affect *C. elephas* infestation.

## 5. Conclusions

Monitoring traps baited with species-specific sex pheromones have played a critical role in pest management since the 1970s, and are commonly used for monitoring insect pests, thereby allowing control measures to be optimally timed [27]. Our research has shown that pheromone-based sampling can be highly efficient for detecting and monitoring tortrix moth populations in chestnut groves, although additional research is needed to provide a proper assessment of population impact. In particular, specific further investigations should focus on the influence of climatic conditions, chestnut variety, harvest method, and fruit size to evaluate their influence on the impact of the chestnut tortrix moths. All data acquired shed light on the presence and abundance of these species in different Italian regions, and these findings are relevant for improving environmentally friendly control strategies.

## Figures and Tables

**Table 1:** Sexual pheromone lures tested in northwestern Italy for monitoring *Pammene fasciana* (L.), *Cydia fagiglandana* (Zeller), and *Cydia splendana* (Hübner) in the two-year period 2018–2019.

Species	Composition	Loading	Notes
*P. fasciana*	Z8-12:Ac + Z8-12:OH	0.75 mg + 0.25 mg	Commercial
*C. fagiglandana*	E8,E10-12Ac	0.01 mg	Commercial
*C. fagiglandana*	E8,E10-12Ac	1.0 mg	Experimental
*C. splendana*	E8,Z10-12:Ac	1.0 mg	Commercial
*C. splendana*	Tricarbonyl-[(8,9,10,11-η)-8,10-dodecadien-1-yl acetate]-iron ^(1)^	2.5 mg	Experimental

^(1)^ Streinz et al. [20].

**Table 2:** Total number and percentage of males captured (across traps and lures) using sexual pheromone baited-traps in the two-year period 2018–2019 (PF, *Pammene fasciana* (L.); CF, *Cydia fagiglandana* (Zeller); CS, *Cydia splendana* (Hübner); CP, commercial pheromone; EP, experimental pheromone).

**2018**												
**Species**	**Pheromone**	**Average No. Males**			**Total No. Males Trapped**	**%**	**Peveragno**	**Villarfocchiardo**	**Carro**	**Montese**	**Badia del Borgo**	**Molazzana**
		**per Site (± SE)**										
PF	CP	503.17	±154.57		3019	84.14	86	1088	602	471	671	101
CF	CP	72.50	±27.83	a	435	12.12	2	38	108	128	159	0
CF	EP	6.00	±2.86	b	36	1.00	2	17	0	12	5	0
CS	CP	3.67	±2.54	A	22	0.61	0	3	0	3	16	0
CS	EP	12.67	±8.31	B	76	2.12	1	23	0	50	2	0
**Total**					3.588							
**2019**												
**Species**	**Pheromone**	**Average No. Males**			**Total No. Males Trapped**	**%**	**Peveragno**	**Villarfocchiardo**	**Carro**	**Montese**	**Badia del Borgo**	**Molazzana**
		**per Site (± SE)**										
PF	CP	86.11	±20.73		5298	76.96	158	1132	2.397	0	793	818
CF	CP	64.83	±12.39	a	389	5.65	1	16	94	0	234	44
CF	EP	61.00	±8.77	a	366	5.32	16	26	124	1	169	30
CS	CP	26.33	±6.75	A	158	2.30	8	15	108	0	17	10
CS	EP	112.17	±47.41	B	673	9.78	11	90	469	4	97	2
**Total**					6.884							

Means followed by different letters are significantly different (Student *t*-test, *p* < 0.05).

**Table 3:** Total number of chestnut husks collected per site and region, and percentage of healthy and damaged husks recorded in the two-year period 2018–2019.

Site	Region	2018			2019		
		Total No. Chestnut Husks	Healthy (%)	Damaged (%)	Total No. Chestnut Husks	Healthy (%)	Damaged (%)
Peveragno	Piedmont	-	-	-	538	51.5	48.5 a
Villar Focchiardo		100	92.0	8.0 a	585	26.2	73.8 b
Carro	Liguria	-	-	-	190	18.4	81.6 b
Montese	Emilia Romagna	161	80.7	19.3 b	-	-	-
Badia del Borgo	Tuscany	120	66.7	33.3 c	-	-	-
Molazzana		-	-	-	-	-	-

-: newly formed chestnut husks not collected. Column means followed by a different letter were significantly different (Tukey’s test, *p* < 0.05).

**Table 4:** Total number of chestnut fruits collected per site and region, and percentage of damage recorded at collection and after storage in the two-year period 2018–2019.

Site	Region	2018				2019			
		Total No. Chestnut Fruit	Damage (%) at Collection	Damage (%) after Rearing	Average No. Larvae Recorded (± SE)	Total No. Chestnut Fruit	Damage (%) at Collection	Damage (%) after Rearing	Average No. Larvae Recorded (± SE)
Peveragno	Piedmont	1000	1.00	1.70 a	2.14 ± 0.98 a	1657	2.66	21.12 a	6.25 ± 1.61 a
Villar Focchiardo		1000	10.20	18.70 b	1.40 ± 0.26 a	1593	4.71	9.42 b	16.11 ± 3.68 b
Carro	Liguria	1169	19.80	22.90 b	5.29 ± 1.33 b	830	8.67	18.43 a	7.36 ± 1.74 a
Montese	Emilia Romagna	1012	4.50	4.60 a	2.44 ± 1.06 a	898	6.68	10.90 b	6.42 ± 1.37 a
Badia del Borgo	Tuscany	2975	2.80	4.60 a	1.10 ± 0.16 a	1156	2.42	9.08 b	3.45 ± 0.89 a
Molazzana		995	8.20	8.70 a	2.27 ± 0.83 a	-	-	-	-

-: chestnut fruits not collected. Column means followed by a different letter were significantly different (Tukey’s test, *p* < 0.05).

## Supplementary Materials

The following are available online at https://www.mdpi.com/2075-4450/11/11/807/s1, Figure S1: Location of study sites in northern Italy used for monitoring the population dynamics of the three chestnut tortricid species *Pammene fasciana* (L.), *Cydia fagiglandana* (Zeller), and *Cydia splendana* (Hübner), Table S1: Sampling sites monitored in the present study.
