# Supplementary material for: Performance of Pheromone-Baited Traps to Monitor the Seasonal Abundance of Tortrix Moths in Chestnut Groves
Source: Insects. 2020 Nov 17;11(11):807. doi: 10.3390/insects11110807 (PMC7697762; doi:10.3390/insects11110807)
Supplement: Supplementary file 1 [file insects-11-00807-s001.pdf]

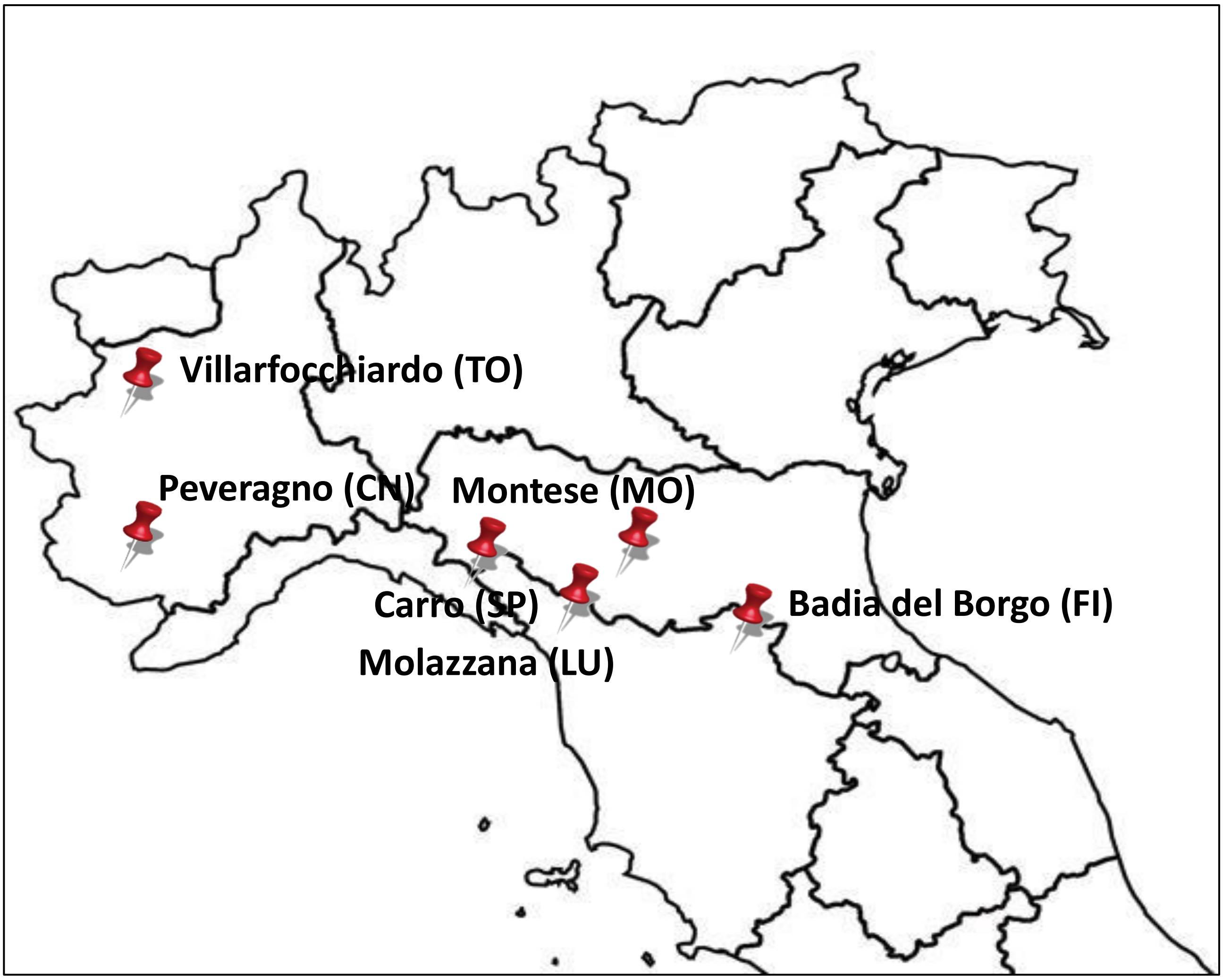

Figure S1. Location of study sites in northern Italy used for monitoring the population dynamics of the three chestnut tortricid species *Pammene fasciana* (L.), *Cydia fagiglandana* (Zeller), and *Cydia splendana* (Hübner).

Table S1. Sampling sites monitored in the present study.

Site	Province	Region	Geographic Coordinates		Altitude (a.s.l.)
			N	E	
Peveragno	Cuneo	Piedmont	44°30'49.4''	07°62'71.3''	860 m
Villar Focchiardo	Torino		45°11'24.7''	07°22'57.1''	450 m
Carro	La Spezia	Liguria	44°06'00.6''	09°46'13.3''	413 m
Montese	Modena	Emilia-Romagna	44°19'06.1''	07°33'18.3''	857 m
Badia del Borgo	Firenze	Tuscany	44°04'52.5''	11°35'19.7''	331 m
Molazzana	Lucca		44°06'40.6''	11°37'52.5''	492 m
